# Marriage and Post-stroke Aphasia: The Long-Time Effects of Group Therapy of Fluent and Non-fluent Aphasic Patients and Their Spouses

**DOI:** 10.3389/fpsyg.2020.01574

**Published:** 2020-07-07

**Authors:** Anna Rasmus, Edyta Orłowska

**Affiliations:** ^1^Instytut Psychologii, Uniwersytet Kazimierza Wielkiego w Bydgoszczy, Bydgoszcz, Poland; ^2^Instytut Psychologii, Uniwersytet Gdański, Gdańsk, Poland

**Keywords:** aphasia, marital relations, couples, therapy effects, caregivers

## Abstract

**Background:**

Studies of therapy influence on after-aphasia marital relations are lacking. Much needs to be learned about the range of factors associated with couples benefiting from therapy. Understanding these issues is key to facilitating optimal post-aphasia outcomes from the perspective of the patient and his caretaking spouse. This paper reports an evaluation of a group therapy intervention conducted with aphasic people and their life partners.

**Methods:**

The intervention comprised of 10 sessions of approximately 90 min duration and included two groups of couples, with fluent and non-fluent aphasic partner. The therapy program consisted of basic communication activities within the group which encouraged sharing of personal experience but mostly relied on psychoeducation, gaining knowledge about after-stroke aphasia. The respondents were interviewed and completed neuropsychological assessment. Quality of marriage was determined using Dyadic Adjustment Scale. Marital adjustment was measured twice, before intervention and after 6 months. Long-time effects of therapy included a significant mean difference in quality of marriage between therapy attendants and controls. Marital relationship decline seems to be worse amongst control subjects, who were not involved in any kind of psychological support. In spite of initial non-distressed relationship they report deterioration of their bond in half a year’s time. We also showed changes in dynamics of quality of marriage during this time in all investigated groups. The implications of these findings for counseling services are discussed.

## Introduction

There is extensive literature on aphasia and it’s various every-day life consequences. As an acquired language disorder, most commonly caused by a stroke, it falls under a definition of a stress related crisis. Experiencing a sudden and unpredicted life event can impair or entirely destroy the ability to continue the tasks related to family cycle and its development (Carter and McGoldrick, 1988). Very early in time researchers tried to apply a crisis model to explain family reactions to aphasia (Webster and Newhoff, 1981). In consequence, many studies proved destructive influence of aphasia on family functioning (Pound et al., 1999; Northcott et al., 2016).

Amongst the factors destabilizing the family system the loss or change of life roles, changes in everyday activities and general emotional crisis are most commonly mentioned (Masterson-Algar et al., 2018). Aphasia impairs functioning in previous roles both occupational and as a family member (Brady et al., 2011; Martinsen et al., 2012). This shift changes the system dynamics and is often a cause of tension. It burdens the marital relations and inhibits behaviors based on mutual reciprocity (Carlsson et al., 2007). Patients may have difficulty with accepting help and families struggle with coping with many short and long term consequences of taking care of aphasic patient (Brady et al., 2011; Martinsen et al., 2012; Adamit et al., 2015). Family members reactions vary from overprotection to depreciation. Most researchers agree, however, that dyadic relationship after the illness onset is no longer based on partnership (Dalemans et al., 2010; Anderson and Whitfield, 2013; Pertl et al., 2019).

Of course, it would be unfounded to claim, that after-aphasia changes in dyadic bond between spouses are always identical as there are many factors influencing quality of marriage. Godwin et al. (2011) note the marked variability of separation and divorce rates across even large sample studies, ranging from fifteen to seventy-eight per cent. Authors highlight qualitatively distinct problems experienced by couples post-injury who do remain together. Therefore our study focuses on couples with initial positive relationship and follows the changes in their marriage evaluations after aphasia’s onset.

Some research indicate important role of aphasia’s type both in general quality of life and quality of marital relationship changes caused by the illness. Achten et al. (2012) found that patients with Broca and mixed non-fluent aphasia reported worse quality of life than patients with anomic aphasia. As there are few reports on this kind of long-term changes in this aspects, this hypothesis still requires empirical verification (Ellis and Peach, 2017). In our previous studies we found aphasias type to be a significant factor in marital communication changes (Orłowska, 2012). The relation between aphasia’s type and marital relationship still requires scientific investigation, therefore this issue has been addressed in our recent study.

Changes in family relations of aphasic patients have to be considered as dynamic and dependent on time factor. One, because of individual progress in rehabilitation of each patient and two, because of general phases of illness. Literature often presents basic aphasia’s stage division including: (1) acute phase (including patient’s hospitalization) (2) intensive speech rehabilitation phase and (3) chronic phase (including patient’s discharge and retuning home). Each stage has its own emotional and organizational consequences and may be connected with different goals. As such marital relationship changes also may fluctuate. It seems, that during first months after hospitalization aphasic patients family confronts lack of control over overall situation. After that fighting with illness stops being perceived as a challenge. Also rehabilitation effects can be seen as to slow and far from the previous image. This disparity reveals actual deficits in every day functioning and generates stress (Simmons-Mackie et al., 2010). In result, family system’s crisis is most visible after 4–6 months (Pa̧chalska, 1999; Orłowska and Jodzio, 2018).

There are many propositions on how to address aphasia’s various consequences for families in therapy. Some researchers addressed this issue inquiring about goals family members have for themselves before therapy. Amongst others, most commonly reported were: (1) to maintain their relationship with the person with aphasia, (2) to be given information, (3) to be given support (Sherratt et al., 2011; Howe et al., 2012). As meta-analysis reports suggest, therapy interventions for aphasic patients and their spouses in social context usually take one of three forms (Simmons-Mackie et al., 2010). First of all it can be a typical training facilitating new resources and strategies of communication between the aphasic patient and his/her partner. Second form addresses psychosocial consequences of aphasia (for example depression, anxiety, and isolation) and engages general psychological counseling (Haun et al., 2008). Third form includes educational programs based on sharing knowledge about aphasia, different behaviors connected with this illness and possible difficulties a patient may encounter in daily life. This form was proved to be connected with improving family functioning of after-stroke and aphasic patients early on (Evans et al., 1988). Most research evaluating therapy effects concentrates on improvement in communication or lessening psychological burden, excluding the actual relationship between spouses (Thompson et al., 2002; Simmons-Mackie et al., 2010). As above mentioned therapy approaches are very different, it is difficult to compare their effectiveness. Nevertheless most studies indicate improvement, both in spouse’s and patient’s functioning, when daily life activities, communication and other psychosocial aspects are considered. Sadly communication therapies effects seem more long-lasting while psychosocial interventions improves functioning during therapy and shortly after (Brown et al., 2010; Anderson and Whitfield, 2013). Little is known about effects of therapy on marital relationship as most studies focus on benefits in general psychosocial and physical wellbeing and quality of life and rarely include the perspective of aphasic patients (Cheng et al., 2014). There is some evidence on needs of family members being related to aphasias time post-onset. At the onset of aphasia, during hospitalization family members rated information about aphasia as most important, followed by psychosocial support and hopefulness (Avent et al., 2011). As these goals are in accordance with those reported by people with aphasia (Worall et al., 2011) we decided to choose education-oriented approach with the element of support-giving and couples therapy. According to Family Systems Theory it is important to identify exchanges of behavior that take place in a given moment of interaction between members of the family or dyad. Those patterns of interaction that spouses maintain, and perpetuate can sustain problematic behavior and ineffective communication (Johnson and Ray, 2016). This aspect of family interaction is especially important in the communication-based systems approach (Watzlawick et al., 1967). As people are constantly trying to define the nature of their relationship attending group therapy meetings for couples experiencing aphasia related difficulties may be beneficial to understanding and changing their communication patterns. In addition group therapy can lead to significant increase in personal resources (Yalom and Leszcz, 2005) which add to family potential, useful in adapting to life after-stroke. Consequently this kind of intervention can lead to discharging tension in marital relationship and more effective communication between spouses.

### Research Aims

There has been limited evaluation of group therapy and its influence on quality of marriage of aphasic couples. Therefore our current study aims to evaluate the effects and applicability of group therapy for couples, based on psychoeducation and social support, on marital adjustment. This research also illustrates the role of aphasia’s type, being in the position of care giver/receiver and time factor in order to grasp long-term effects of applied intervention. Specifically, we tested one hypothesis derived based on available literature and our previous research there is a significant mean difference in quality of marriage between therapy attendants and controls after 6 months time.

We also state an explorative questions: (1) Is there a significant change in dynamics of changes in quality of marriage during 6 months in all investigated groups?; (2) Are there any aspects of marriage adjustment that benefit from therapy more than others?

## Materials and Methods

### Sample

Research data used in this program evaluation study was regularly collected and archived as a means of evaluating treatment efficacy. The research was conducted initially at 3 Polish neurology hospital in-wards over the time of 3 years. The respondents were selected amongst patients recently experiencing after-stroke aphasia and their spouses. These patients were in-patients at the time of the initial interview. Patients after one left hemisphere stroke with mild or moderate fluent or non-fluent aphasia as confirmed in the Cracow Neurolinguistic Aphasia Battery CNAB (Pachalska et al., 1995) (>75 Aphasia Quotient, AQ) and medical investigation. All participants had to able to comprehend questions and indicate their consent. They also had to live within the local urban/exurban area for the purposes of attending group therapy and follow-up. Participants were informed of the nature and purpose of gathering treatment outcome measures, noting the use of the collected data would assist in providing effective of other aphasic couples in the future. The importance of their consent was stressed as permission to use the collected data in published research studies was requested and received from the subjects. Participants were assured that their confidentiality would be maintained throughout the archival and evaluation process. From the initial cohort of 150 couples 96 confirmed interest in therapy. Later 16 initially selected couples resigned during first 2 weeks of treatment, due to external factors. Finally four experimental groups have been randomly selected out of 80 couples, each comprising of 20 subjects: (1) patients with fluent aphasia (FAP) (2) spouses of patients with fluent aphasia (FAPS) (3) patients with non-fluent aphasia (NFAP) (4) spouses of patients with non-fluent aphasia (NFAPS).

Control subjects were recruited in similar fashion amongst 54 couples (in-patients and their spouses) not interested in therapy or otherwise not able to attend the meetings due to external factors. Suitable sampling was conducted in order to match control and experimental group subjects considering basic factors like: gender, education, age, and aphasia type.

All the respondents were married with the mean of the duration of marriage of 19.26 years, with no previous divorce record.

#### Exclusion Criteria

In order to capture the marital changes related to experiencing aphasia couples with initial marital problems were eliminated from the study. Using criteria described by Jacobson et al. (1984) couples whose general Dyadic Adjustment Scale (DAS) score was equal to or less than 97 were classified as martially distressed, whereas couples whose DAS scores were greater than 97 before therapy were classified as martially non-distressed and were excluded from further examination. Patients were also disqualified if they previously experienced brain injury or stroke, suffered from dementia or other neurological disease before or during our investigation. All subjects were confirmed to not suffer from severe mental problems like depression, anxiety, drugs, or alcohol abuse or family violence. In order to eliminate severe mental health problems authors used clinical interview, medical history analysis, as well as Modified Hospital Anxiety and Depression Scale HADS-M (Walden-Gałuszko and Majkowicz, 2000) with the cut-off point of 7. Due to communication difficulties all patients with severe aphasia were considered as not suitable and as such not considered in this study.

Analyses of variance (ANOVAs) revealed no significant differences between therapy groups and controls in age education, number of years married or initial quality of marriage. All respondents were married with the mean of the duration of marriage of 19.26 years and expressed consent by desire. Respondents basic characteristics and demographic data are presented in Table 1.

**TABLE 1:** Demographic characteristics of patients and their spouses in therapy and control groups.

	Therapy (*n* = 80)				Controls (80)			
	FAP (*n* = 20)	FAPS (*n* = 20)	NFAP (*n* = 20)	NFAPS (*n* = 20)	FAP (*n* = 20)	FAPS (*n* = 20)	NFAP (*n* = 20)	NFAPS (*n* = 20)
**Gender**								
Male	11	9	14	6	11	9	14	6
Female	9	11	6	14	9	11	6	14
**Age**								
*M*	56.5	51.5	54.25	55.00	54.2	52.2	53.40	52.9
*SD*	3.22	5.64	6.44	6.92	5.29	6.0	5.35	6.07
**Education**								
*M*	13.05	12.85	13.40	11.85	12.30	13.10	14.00	13.15
*SD*	2.92	2.62	2.48	1.95	3.58	3.58	2.63	2.85

*M, mean; SD, standard deviation; FAP, fluent aphasic patients; FAPS, fluent aphasic patients spouses; NFAP, non-fluent aphasic patients; NFAPS, non-fluent aphasic patients spouses.*

### Procedure

In our study a double assessment procedure was applied. The first examination was carried out during hospitalization within 3 weeks after the illness onset. The second examination took place 6 months after the first, and was conducted at the patient’s home environment.

The therapy comprised of 10 once a week sessions of approximately 90 min duration and included two separate groups of couples, with fluent and non-fluent aphasic partner. The program was derived from classic guidebooks, especially “Understanding aphasia” (Pa̧chalska, 1993) and based on Aphasia Couples Therapy (ACT) encouraging a group therapy form (Boles and Lewis, 2001). We decided to combine education-oriented approach with the element of support-giving and couples therapy (Boles and Lewis, 2001; Boles, 2006, 2009, 2011). First two meetings were dedicated to educating couples on aphasia. Later sessions were less direct and unstructured and followed sociolinguistic and family system therapy guidelines. Group therapy was conducted by both experienced speech therapist and family therapist previously working with aphasic patients. During sessions elements of Aphasia Couples Therapy (ACT) Workbook were incorporated, especially those solution focused and showing different perspectives of both healthy and aphasic partners. Group therapy is not a novel solution and has been used in aphasia intervention for decades (Revenson et al., 2016). It is a preferred form of treatment in many approaches (Kagan, 1995). Its advantages include interactive communication, peer interaction, and conversational practice. Additionally all group intervention provide therapeutic factors such as giving hope, versatility and widening support network (Yalom and Leszcz, 2005). It has been proved to be effective to involve significant others in this kind of interventions. Usually caregivers, spouses and other close relatives of people with aphasia are invited to attend (Boles, 2006, 2009, 2011; Turner and Whitworth, 2006; Kagan et al., 2008; Brown et al., 2010; Anderson and Whitfield, 2013).

### Measures

All in-patients were diagnosed by a neurologist, speech therapist to have aphasia. In order to complement the medical diagnosis we administered Cracow Neurolinguistic Battery of Aphasia (CNBAE).

All participants had been interviewed, screened with Mini Mental State Examination (MMSE, with cutoff <24 points) for dementia and diagnosed with Hospital Anxiety and Depression Scale (HADS) in order to determine levels of anxiety and depression. Its psychometric proprieties were proven to be acceptable, also on Polish in-patients. Using the cut-off value ≥7 points, the results on post-stroke Polish population were as following: depression subscale – sensitivity: 90.0%, specificity 92.2%, anxiety subscale: sensitivity: 86.5%, specificity 94.9%, which was the most optimal cut-off point. Cronbach α: for the depression subscale was 0.892, for the anxiety subscale was 0.815.

All subjects after initial evaluation were approached and asked to provide information about their relationship with the partner.

Dyadic Adjustment Scale (DAS; Spanier, 1976; Cieślak, 1989) was administered at pre-treatment and post-treatment (after 6 months) to measure of marital quality of life of both healthy spouses and their aphasic partners. This self-report scale is divided into 4 subscales : (1) Dyadic Consensus – degree to which respondent agrees with partner; (2) Dyadic Satisfaction – degree to which respondent feels satisfied with partner; (3) Dyadic Cohesion –degree to which respondent and partner participate in activities together; (4) Affectional Expression –degree to which respondent agrees with partner regarding emotional affection. Summing the scores for all four subscales yields a total dyadic adjustment score. DAS has been shown to have high internal consistency, discriminant efficiency. Compared with other measures of global marital satisfaction it is more sensitive to treatment effects (Whisman and Jacobson, 1992). It has been previously used on similar cohorts (Łapkiewicz et al., 2008; Kieffer-Kristensen and Teasdale, 2011; Ghedin et al., 2017).

Dyadic Adjustment Scale has also been used in numerous studies to examine marital satisfaction in older adults (Carr et al., 2000; O’Rourke, 2005; Yorgason et al., 2006; Garand et al., 2007). It has demonstrated excellent reliability with a total scale Cronbach’s coefficient alpha of 0.96 (Spanier, 1976). In addition, other investigators have reported comparable values with subscale internal consistency reliabilities ranging from 0.73 to 0.92 for Dyadic Consensus, 0.77 to 0.94 for Dyadic Satisfaction, 0.58 to 0.73 for Affectional Expression, and 0.72 to 0.86 for Dyadic Consensus (Spanier, 2001). Measures of content validity, criterion-related validity, concurrent and predictive validity, and convergent validity also support the strength of the DAS in measuring the constructs that it purports to assess (Spanier, 1976, 2001). A recent meta-analysis of the reliability of DAS involving 91 studies that included 128 samples consisting of 25,035 participants (Graham et al., 2006) revealed a mean alpha for the total score of 0.92.

### Statistical Analysis

Statistica version 13.1 program was used to analyze the gathered data. We used *two-factor repeated-measures analysis of variance* to analyze whether the therapy attendance and aphasia’s type influence marital adjustment of aphasic patients and their care-giving spouses. Time was an intra-object factor measured on two levels (pretest-before the therapy and posttest- 6 months after). Dependent variable was the quality of marriage, measured with Dyadic Adjustment Scale (general score, and four subscales: marital consensus, cohesion, satisfaction and affectional expression). Analysis of variance was conducted in multivariate model. If a main effect of aphasia’s type, therapy or time (pretest and posttest) or an inter− action was found, *post hoc* analysis was carried out for designed group differences on DAS scores. *Post hoc* pairwise comparisons consisted of Bonferroni test. Hypothesis tests used α = 0.05 as the criterion for significant effects.

Our hypothesis states that there is a significant mean difference in quality of marriage between therapy attendants and controls after 6 months time. As mentioned before, our previous studies show, that experiencing fluent or non-fluent aphasia has different consequences for marital adjustment and it is also important for marital bond if a spouse is a healthy care-giver or aphasic care receiver. In order to control those variables that can influence the main effect of therapy we decided to create four criteria groups. As such our investigated variables include:

– Dependent variable: marital adjustment.
– Independent variable: group therapy attendance.
– Controlled variables:
  • Aphasia’s type experienced by one of the spouses (fluent or non-fluent).
  • The role in the relationship after stroke (being an aphasic patient or caregiving spouse).

In order to show effect of therapy in each four criteria groups we compared marital adjustment scores of fluent aphasic patients attending and not attending therapy (FAP therapy and control groups), non-fluent aphasic patients attending and not attending therapy (NFAP), spouses of people experiencing fluent aphasia attending and not attending therapy (FAPS) and spouses of people experiencing non-fluent aphasia attending and not attending therapy, both in pretest and posttest. We used paired *t*-student test to show those differences.

As we also state an explorative questions concerning dynamics of changes in quality of marriage in time during 6 months in and aspects of marriage adjustment that benefit from therapy we analyzed within-group changes in marital adjustment level comparing pretest and posttest DAS score in each investigated group. We decided on running this analyses separately for each criteria group (FAP, NFAP, FAPS, and NFAPS) with separation of subjects attending and not attending therapy (therapy and controls). We used online calculator to illustrate size effect of statistically significant differences between compared groups or test–retest scores with Cohen’s *d* values.

## Results

The relationship between aphasia’s type, therapy attendance, time factor general Dyadic Adjustment Scale scores in patients population is presented in Table 2. For DAS total score there was a main effect for time and therapy attendance [*F*(1,76) = 5.01; *p* = 0.028; $\eta_{\text{p}}^{2}$ = 0.06]. As *post hoc* comparisons using the Bonferroni test indicate, this concerns only fluent aphasic patients not included in intervention (FAP controls). The mean general score in DAS for FAP controls in pretest (*M* = 107.3, *SD* = 14.80) was significantly different (*p* = 0.029), *d*_*Cohen*_ = 0.66 than general DAS score for FAP controls in posttest (*M* = 93.20; *SD* = 26.08). However, there was no other significant differences for other groups in time.

**TABLE 2:** ANOVA and Bonferroni corrected *post hoc t*-test results for general Dyadic Adjustment Scale scores of people with aphasia.

	ANOVA					*Post hoc* comparisons		
	df	Mean square	*F*	*p*	Eta-square	Between group comparisons	*p*	Effect size
Aphasia type	1	7924	26.600	**<0.001**	0.059	FAP therapy 1 vs. FAP controls 1	1.000	
Therapy	1	469	1.575	0.213	0.020	FAP therapy 2 vs. FAP controls 2	0.244	
Aphasia type × therapy	1	130	0.435	0.511	0.006	NFAP therapy 1 vs. NFAP controls 1	1.000	
						NFAP therapy 2 vs. NFAP controls 2	1.000	

						**Pretest and posttest comparisons**		

Time	1	1891	10.948	**0.001**	0.126			
Time × aphasia type	1	6	0.037	0.848	0.037	FAP therapy 1 vs. FAP therapy 2	1.000	
Time × therapy	1	865	5.008	**0.028**	0.062	FAP controls 1 vs. FAP controls 2	**0.029**	
Time × therapy × aphasia	1	366	2.119	0.150	0.027	NFAP therapy 1 vs. NFAP therapy 2	1.000	
						NFAP controls 1 vs. NFAP controls 2	0.992	

*Aphasia’s type, refers to fluent vs. non-fluent aphasia; therapy, refers to group therapy intervention; time, refers to pretest and posttest assessment; F, F-test value; p, significance; effect size, as measured by Cohen’s d; FAP therapy, fluent aphasic people attending therapy; FAP controls, fluent aphasic people not attending therapy, NFAP therapy, non-fluent aphasic people attending therapy; NFAP controls, non-fluent aphasic people not attending therapy; 1, first assessment (pretest); 2, second assessment (posttest). We bolded statistical significance(significant p level).*

The relationship between aphasia’s type, therapy attendance, time factor general Dyadic Adjustment Scale scores provided by caregiving spouses of aphasic people is showed in Table 3. For DAS total score there was a main effect for therapy attendance [*F*(1,76) = 8.85; *p* = 0.004, $\eta_{\text{p}}^{2}$ = 0.10]. *Post hoc* comparisons using the Bonferroni test indicated no significant differences between groups chosen according to our hypothesis. Nevertheless there were also two effects for aphasias type in interaction with time [*F*(1,76) = 21.99; *p* < 0.001; $\eta_{\text{p}}^{2}$ = 0.22] and therapy attendance interacting with time [*F*(1,76) = 12.96, *p* < 0,001, $\eta_{\text{p}}^{2}$ = 0.15]. Bonferroni *post hoc* test revealed the FAPS controls general scores in pretest (*M* = 107.50; *SD* = 19.45) were higher (*p* < 0.001, *d_*Cohen*_* = 0.82) than in posttest (*M* = 91.60, *SD* = 19.25). Similarly, NFAPS therapy general DAS scores significantly dropped in time (*p* < 0.001; *d*_*Cohen*_ = 1.89) (from *M* = 120.15, *SD* = 10.54 to *M* = 101.00, *SD* = 9,68). Spouses of non-fluent aphasic people not attending therapy (NFAPS controls) also evaluated general marital adjustment significantly higher (*p* < 0,001, *d_*Cohen*_* = 1,49) initially (*M* = 114.95, *SD* = 14.84) than in second assessment after 6 months (*M* = 86.70, *SD* = 22.59).

**TABLE 3:** ANOVA and Bonferroni corrected *post hoc t*-test results for general Dyadic Adjustment Scale scores of care-giving spouses of aphasic people.

	ANOVA					*Post hoc* comparisons		
	df	Mean square	*F*	*p*	Eta-square	Between group comparisons	*p*	Effect size
Aphasia type	1	69	0.162	0.688	0.002	FAPS therapy 1 vs. FAPS controls 1	1.000	
Therapy	1	3754	8.852	**0.004**	0.104	FAPS therapy 2 vs. FAPS controls 2	0.07	
Aphasia type × therapy	1	0	0.001	0.978	0.001	NFAPS therapy 1 vs. NFAPS controls 1	1.000	
						NFAPS therapy 2 vs. NFAPS controls 2	0.156	

						**Pretest and posttest comparisons**		

Time	1	11273	130.627	**<0.001**	0.632			
Time × aphasia type	1	1898	21.988	**<0.001**	0.224	FAPS therapy 1 vs. FAPS therapy 2	1.000	
Time × therapy	1	1118	12.959	**<0.001**	0.146	FAPS controls 1 vs. FAPS controls 2	**<0.001**	0.82
Time × therapy × aphasia	1	23	0.269	0.605	0.003	NFAPS therapy 1 vs. NFAPS therapy 2	**<0.001**	1.89
						NFAPS controls 1 vs. NFAPS controls 2	**<0.001**	1.47

*Aphasia’s type, refers to fluent vs. non-fluent aphasia; therapy, refers to group therapy intervention; time, refers to pretest and posttest assessment; F, F-test value; p, significance; effect size, as measured by Cohen’s d; FAPS therapy, spouses of fluent aphasic people attending therapy; FAPS controls, spouses of fluent aphasic people not attending therapy; NFAPS therapy, spouses of non-fluent aphasic people attending therapy; NFAPS controls, spouses of non-fluent aphasic people not attending therapy; 1, first assessment (pretest); 2, second assessment (posttest). We bolded statistical significance(significant p level).*

The relationship between aphasia’s type, therapy attendance, time factor Dyadic Adjustment Scale in consensus scores provided by aphasic patients as reflected by ANOVA results is presented in Table 4. Data analysis showed significant effect for interaction of aphasia’s type and therapy attendance *F*(1,76) = 4,68; *p* = 0,034; $\eta_{\text{p}}^{2}$ = 0,06. *Post hoc* comparisons using the Bonferroni test indicated no significant differences between groups except fluent aphasic patients (FAP) in second assessment. Fluent aphasic patients attending the meetings evaluate marital consensus (*p* = 0.009; *d*_*Cohen*_ = 1.00) higher (*M* = 49.75, *SD* = 8.04) than those not involved in therapy (*M* = 40.80, *SD* = 9,74). There were also significant effects of interaction of time and therapy attendance [*F*(1,76) = 10,79, *p* = 0,001, $\eta_{\text{p}}^{2}$ = 0,12] and time, therapy attendance and aphasia’s type [*F*(1,76) = 7,16; *p* = 0,009; $\eta_{\text{p}}^{2}$ = 0,09]. *Post hoc* Bonferroni test revealed fluent aphasic patients not involved in therapy (FAP controls) evaluate marital consensus in posttest significantly (*p* < 0.001, *d*_*Cohen*_ = 1.21) lower (*M* = 40.80, *SD* = 9.74) compared with their initial assessment (*M* = 52.60, *SD* = 9.72).

**TABLE 4:** ANOVA and Bonferroni corrected *post hoc t*-test results for Dyadic Adjustment Scale consensus scores of people with aphasia.

	ANOVA					*Post hoc* comparisons		
	df	Mean square	*F*	*p*	Eta-square	Between group comparisons	*p*	Effect size
Aphasia type	1	2272.6	27.033	**<0.001**	2.262	FAP therapy 1 vs. FAP controls 1	1.000	
Therapy	1	7.7	0.091	0.764	0.001	FAP therapy 2 vs. FAP controls 2	**0.009**	1.001
Aphasia type × therapy	1	4.393.8	4.684	**0.034**	0.058	NFAP therapy 1 vs. NFAP controls 1	1.000	
						NFAP therapy 2 vs. NFAP controls 2	1.000	

						**Pretest and posttest comparisons**		

Time	1	1107.8	34.06	**<0.001**	0.309			
Time × aphasia type	1	54.1	1.662	0.201	0.021	FAP therapy 1 vs. FAP therapy 2	1.000	
Time × therapy	1	351.1	10.794	**0.001**	0.124	FAP controls 1 vs. FAP controls 2	**<0.001**	1.213
Time × therapy × aphasia	1	232.8	7.158	**0.009**	0.086	NFAP therapy 1 vs. NFAP therapy 2	1.000	
						NFAP controls 1 vs. NFAP controls 2	0.332	

*Aphasia’s type, refers to fluent vs. non-fluent aphasia; therapy, refers to group therapy intervention; time, refers to pretest and posttest assessment; F, F-test value; p, significance; effect size, as measured by Cohen’s d; FAP therapy, fluent aphasic people attending therapy; FAP controls, fluent aphasic people not attending therapy; NFAP therapy, non-fluent aphasic people attending therapy; NFAP controls, non-fluent aphasic people not attending therapy; 1, first assessment (pretest); 2, second assessment (posttest). We bolded statistical significance(significant p level).*

Results of similar analysis for care-giving spouses population concerning marital consensus (Table 5.) show significant effect for therapy [*F*(1,76) = 4.05, *p* = 0.048, $\eta_{\text{p}}^{2}$ = 0.05] and interaction of therapy and aphasia’s type [*F*(1,76) = 13.06, *p* < 0.001, η^2^ = 0.15]. *Post hoc* Bonferroni *t*-test indicated, that spouses of fluent aphasic people attending therapy (FAPS therapy) evaluate marital consensus (*p* = 0.001, *d*_*Cohen*_ = 1.75) higher (*M* = 51.65, *SD* = 5.14) than controls (*M* = 41.60, *SD* = 6.28) in second assessment. There was also significant effect of interaction of time with aphasia’s type [*F*(1,76) = 10.36, *p* = 0.002, $\eta_{\text{p}}^{2}$ = 0.12] and time with therapy attendance [*F*(1,76) = 4.5, *p* = 0.037, η^2^ = 0.06]. As proven in *post hoc* Bonferroni *t*-test, evaluations of subjects from FAPS group, without therapy intervention, drop significantly (*p* = 0.018, *d*_*Cohen*_ = 1.00) over time (pretest *M* = 50.00, *SD* = 9.99 versus posttest *M* = 41.16, *SD* = 6.28). Similarly, NFAPS therapy attendees report significantly lower (*p* < 0.001, *d*_*Cohen*_ = 1.53) consensus scores in time (pretest *M* = 54.90, *SD* = 6.75 versus posttest *M* = 43.90, *SD* = 7.62). This effect can be confirmed also for NFAPS subjects not attending the meetings as their consensus evaluations drop (*p* < 0.001, *d*_*Cohen*_ = 1.33) after 6 months (from *M* = 58.35, *SD* = 6.82 in pretest to *M* = 44.30, *SD* = 13.34 in posttest).

**TABLE 5:** ANOVA and Bonferroni corrected *post hoc t*-test results for Dyadic Adjustment Scale consensus scores of spouses of aphasic people.

	ANOVA					Post hoc comparisons		
	df	Mean square	F	*p*	Eta-square	Between group comparisons	p	Effect size
Aphasia type	1	66.3	1.256	0.266	0.016	FAPS therapy 1 vs. FAPS controls 1	1.000	
Therapy	1	213.9	4.052	**0.048**	0.051	FAPS therapy 2 vs. FAPS controls 2	**0.001**	1.750
Aphasia type × therapy	1	718.3	13.605	**<0.001**	0.152	NFAPS therapy 1 vs. NFAPS controls 1	1.000	
						NFAPS therapy 2 vs. NFAPS controls 2	1.000	

						**Pretest and posttest comparisons**		

Time	1	3036.3	53.115	**<0.001**	0.415			
Time × aphasia type	1	581.4	10.362	**0.002**	0.120	FAPS therapy 1 vs. FAPS therapy 2	1.000	
Time × therapy	1	252.5	4.500	**0.037**	0.056	FAPS controls 1 vs. FAPS controls 2	**0.018**	1.001
Time × therapy × aphasia	1	39.0	0.695	0.407	0.009	NFAPS therapy 1 vs. NFAPS therapy 2	**<0.001**	1.528
						NFAPS controls 1 vs. NFAPS controls 2	**<0.001**	1.326

*Aphasia’s type, refers to fluent vs. non-fluent aphasia; therapy, refers to group therapy intervention; time, refers to pretest and posttest assessment; F, F-test value; p, significance; effect size, as measured by Cohen’s d; FAPS therapy, spouses of fluent aphasic people attending therapy; FAPS controls, spouses of fluent aphasic people not attending therapy; NFAPS therapy, spouses of non-fluent aphasic people attending therapy; NFAPS controls, spouses of non-fluent aphasic people not attending therapy; 1, first assessment (pretest); 2, second assessment (posttest). We bolded statistical significance(significant p level).*

For DAS cohesion subscale score in aphasic subjects assessment, there was a main effect for aphasia’a type [*F*(1,76) = 8.287; *p* = 0.005; $\eta_{\text{p}}^{2}$ = 0.10] (Table 6). For DAS cohesion subscale score reported by spouses of aphasic people (Table 7), there was a main effect for therapy attendance [*F* = 10.02 (1,76), *p* = 0.002, η^2^η^2^ = 0.12]. For DAS satisfaction subscale score (Table 8) there was a main effect for aphasia’s type [*F* = (1.76) = 15.32; *p* < 0.001, $\eta_{\text{p}}^{2}$ = 0,17]. However, for all those effects, *post hoc* comparisons using the Bonferroni test indicated no significant differences between groups chosen according to our hypothesis.

**TABLE 6:** Two-way ANOVA and Bonferroni corrected *post hoc t*-test results for Dyadic Adjustment Scale cohesion scores of people with aphasia.

	ANOVA					*Post hoc* comparisons		
	df	Mean square	*F*	*p*	Eta-square	Between group comparisons	*p*	Effect size
Aphasia type	1	129.60	8.287	**0.005**	0.098	FAP therapy 1 vs. FAP controls 1	1.000	
Therapy	1	40	2.256	0.114	0.033	FAP therapy 2 vs. FAP controls 2	0.133	
Aphasia type × therapy	1	14.40	0.921	0.340	0.012	NFAP therapy 1 vs. NFAP controls 1	1.000	
						NFAP therapy 2 vs. NFAP controls 2	1.000	

						**Pretest and posttest comparisons**		

Time	1	3.03	0.393	0.532	0.005			
Time × aphasia type	1	0.02	0.003	0.955	0.001	FAP therapy 1 vs. FAP therapy 2	0.133	
Time × therapy	1	18.22	2.370	0.128	0.127	FAP controls 1 vs. FAP controls 2	1.000	
Time × therapy × aphasia	1	27.23	3.540	0.064	0.044	NFAP therapy 1 vs. NFAP therapy 2	1.000	
						NFAP controls 1 vs. NFAP controls 2	1.000	

*Aphasia’s type, refers to fluent vs. non-fluent aphasia; therapy, refers to group therapy intervention; time, refers to pretest and posttest assessment; F, F-test value; p, significance; effect size, as measured by Cohen’s d; FAP therapy, fluent aphasic people attending therapy; FAP controls, fluent aphasic people not attending therapy; NFAP therapy, non-fluent aphasic people attending therapy; NFAP controls, non-fluent aphasic people not attending therapy; 1, first assessment (pretest); 2, second assessment (posttest). We bolded statistical significance(significant p level).*

**TABLE 7:** Two-way ANOVA and Bonferroni corrected *post hoc t*-test results for Dyadic Adjustment Scale cohesion scores of spouses of people with aphasia.

	ANOVA					*Post hoc* comparisons		
	df	Mean square	*F*	*p*	Eta-square	Between group comparisons	*p*	Effect size
Aphasia type	1	56.41	1.288	0.260	0.016	FAPS therapy 1 vs. FAPS controls 1	0.909	
Therapy	1	438.91	10.022	0.002	0.116	FAPS therapy 2 vs. FAPS controls 2	1.000	
Aphasia type × therapy	1	1.06	0.0241	0.877	0.001	NFAPS therapy 1 vs. NFAPS controls 1	1.000	
						NFAPS therapy 2 vs. NFAPS controls 2	0.113	

						**Pretest and posttest comparisons**		

Time	1	10.51	0.267	0.607	0.003			
Time × aphasia type	1	10.51	0.2672	0.606	0.003	FAPS therapy 1 vs. FAPS therapy 2	1.000	
Time × therapy	1	1.41	0.0358	0.850	0.001	FAPS controls 1 vs. FAPS controls 2	1.000	
Time × therapy × aphasia	1	15.01	0.3817	0.538	0.005	NFAPS therapy 1 vs. NFAPS therapy 2	1.000	
						NFAPS controls 1 vs. NFAPS controls 2	1.000	

*Aphasia’s type, refers to fluent vs. non-fluent aphasia; therapy, refers to group therapy intervention; time, refers to pretest and posttest assessment; F, F-test value; p, significance; effect size, as measured by Cohen’s d; FAPS therapy, spouses of fluent aphasic people attending therapy; FAPS controls, spouses of fluent aphasic people not attending therapy; NFAPS therapy, spouses of non-fluent aphasic people attending therapy; NFAPS controls, spouses of non-fluent aphasic people not attending therapy; 1, first assessment (pretest); 2, second assessment (posttest).*

**TABLE 8:** Two-way ANOVA and Bonferroni corrected *post hoc t*-test results for Dyadic Adjustment Scale satisfaction scores of people with aphasia.

	ANOVA					*Post hoc* comparisons		
	df	Mean square	*F*	*p*	Eta-square	Between group comparisons	*p*	Effect size
Aphasia type	1	965.3	15.324	**<0.001**	0.168	FAP therapy 1 vs. FAP controls 1	1.000	
Therapy	1	51.8	0.822	0.368	0.011	FAP therapy 2 vs. FAP controls 2	0.798	
Aphasia type × therapy	1	11.6	0.183	0.670	0.002	NFAP therapy 1 vs. NFAP controls 1	1.000	
						NFAP therapy 2 vs. NFAP controls 2	1.000	

						**Pretest and posttest comparisons**		

Time	1	61.3	2.438	0.123	0.003			
Time × aphasia type	1	41.0	1.632	0.205	0.021	FAP therapy 1 vs. FAP therapy 2	1.000	
Time × therapy	1	146.3	5.822	**0.018**	0.071	FAP controls 1 vs. FAP controls 2	1.000	
Time × therapy × aphasia	1	45.2	1.797	0.184	0.023	NFAP therapy 1 vs. NFAP therapy 2	1.000	
						NFAP controls 1 vs. NFAP controls 2	1.000	

*Aphasia’s type, refers to fluent vs. non-fluent aphasia; therapy, refers to group therapy intervention; time, refers to pretest and posttest assessment; F, F-test value; p, significance; effect size, as measured by Cohen’s d; FAP therapy, fluent aphasic people attending therapy; FAP controls, fluent aphasic people not attending therapy; NFAP therapy, non-fluent aphasic people attending therapy; NFAP controls, non-fluent aphasic people not attending therapy; 1, first assessment (pretest); 2, second assessment (posttest). We bolded statistical significance (significant p level).*

There was a significant difference between the groups for the marital satisfaction scores provided by spouses of people with aphasia (Table 9). There were effects for interaction between aphasia’s type and therapy attendance [*F*(1,76) = 4.91, *p* = 0.030, η^2^ = 0.06] and also aphasia’s type and time factor [*F*(1,76) = 29.56; *p* < 0,001; $\eta_{\text{p}}^{2}$ = 0.28]. *Post hoc* Bonferroni test revealed the spouses of non-fluent aphasic patients not attending therapy (NFAPS controls) were less satisfied (*p* < 0.001, *d*_*Cohen*_ = 1.55) with their marriage after 6 months’ time (pretest *M* = 36.70, *SD* = 8.15, posttest *M* = 25.75, *SD* = 5.73). Additionally, spouses of people with non-fluent aphasic people involved in therapy (NFAPS therapy) also evaluated their marital satisfaction significantly lower (*p* = 0.003, *d*_*Cohen*_ = 1.19) in second assessment (pretest *M* = 37.05, *SD* = 5.64 and posttest *M* = 30.25, *SD* = 5.80).

**TABLE 9:** Two-way ANOVA and Bonferroni corrected *post hoc t*-test results for Dyadic Adjustment Scale satisfaction scores of spouses of people with aphasia.

	ANOVA					*Post hoc* comparisons		
	df	Mean square	*F*	*p*	Eta-square	Between group comparisons	*p*	Effect size
Aphasia type	1	21.0	0.244	0.622	0.003	FAPS therapy 1 vs. FAPS controls 1	1.000	
Therapy	1	27.2	0.316	0.575	0.004	FAPS therapy 2 vs. FAPS controls 2	1.000	
Aphasia type × therapy	1	422.5	4.909	**0.030**	0.060	NFAPS therapy 1 vs. NFAPS controls 1	1.000	
						NFAPS therapy 2 vs. NFAPS controls 2	1.000	

						**Pretest and posttest comparisons**		

Time	1	748.2	26.707	**<0.001**	0.26			
Time × aphasia type	1	828.1	29.558	**<0.001**	0.280	FAPS therapy 1 vs. FAPS therapy 2	1.000	
Time × therapy	1	48.4	1.728	0.193	0.022	FAPS controls 1 vs. FAPS controls 2	1.000	1.189
Time × therapy × aphasia	1	38.0	1.357	0.248	0.017	NFAPS therapy 1 vs. NFAPS therapy 2	**0.003**	1.554
						NFAPS controls 1 vs. NFAPs controls 2	**<0.001**	

*Aphasia’s type, refers to fluent vs. non-fluent aphasia; therapy, refers to group therapy intervention; time, refers to pretest and posttest assessment; F, F-test value; p, significance; effect size, as measured by Cohen’s d; FAPS therapy, spouses of fluent aphasic people attending therapy; FAPS controls, spouses of fluent aphasic people not attending therapy; NFAPS therapy, spouses of non-fluent aphasic people attending therapy; NFAPS controls, spouses of non-fluent aphasic people not attending therapy; 1, first assessment (pretest); 2, second assessment (posttest). We bolded statistical significance (significant p level).*

There was a significant difference between the groups for the affectional expression in marriage in assessment of people with aphasia (Table 10). There were two main effects for aphasias type [*F*(1,76) = 10.42; *p* = 0.002; $\eta_{\text{p}}^{2}$ = 0.12] and therapy attendance [*F*(1,76) = 8.51, *p* = 0.005, η^2^ = 0.10]. *Post hoc* Bonferroni test analysis indicated, that FAP therapy subjects evaluated emotional expression in their relationship significantly (*p* = 0.030, *d*_*Cohen*_ = 0.780) higher (*M* = 9.20, *SD* = 1.91) than FAP controls (*M* = 7.40, *SD* = 2.64) in second assessment. NFAP therapy subjects gave higher scores (*M* = 10.77, *SD* = 0.46) in affectional expression (*p* = 0.006, *d*_*Cohen*_ = 1.10) than NFAP controls (*M* = 8.70, *SD* = 2.60). There was an additional interaction effect of time and therapy attendance (*F* = 23.82, *p* < 0.001, $\eta_{\text{p}}^{2}$ = 0.24). *Post hoc* analysis revealed significant (*p* < 0.001, *d*_*Cohen*_ = 1.42) decrease in emotional expression evaluations in FAP controls group in time (from pretest *M* = 10.15, *SD* = 0.74, to posttest *M* = 7.40, *SD* = 2.64). Similarly, NFAP control subjects evaluated affectional expression in their relationship as lower over time (pretest *M* = 10.20, *SD* = 1.32, posttest *M* = 8.70, *SD* = 2.60).

**TABLE 10:** Two-way ANOVA and Bonferroni corrected *post hoc t*-test results for Dyadic Adjustment Scale affectional expression scores of people with aphasia.

	ANOVA					*Post hoc* comparisons		
	df	Mean square	*F*	*p*	Eta-square	Between group comparisons	*p*	Effect size
Aphasia type	1	43.06	10.422	**0.002**	0.121	FAP therapy 1 vs. FAP controls 1	1.000	
Therapy	1	35.16	8.510	**0.005**	0.101	FAP therapy 2 vs. FAP controls 2	**0.030**	0.780
Aphasia type × therapy	1	5.26	1.272	0.263	0.016	NFAP therapy 1 vs. NFAP controls 1	1.000	
						NFAP therapy 2 vs. NFAP controls 2	**0.006**	1.100

						**Pretest and posttest comparisons**		

Time	1	51.76	31.601	**<0.001**	0.293			
Time × aphasia type	1	6.01	3.667	0.059	0.046	FAP therapy 1 vs. FAP therapy 2	1,000	
Time × therapy	1	39.01	23.816	**<0.001**	0.238	FAP controls 1 vs. FAP controls 2	**<0.001**	1.418
Time × therapy × aphasia	1	2.26	1.378	0.0244	0.018	NFAP therapy 1 vs. NFAP therapy 2	1,000	
						NFAP controls 1 vs. NFAP controls 2	**0.012**	0.470

*Aphasia’s type, refers to fluent vs. non-fluent aphasia; therapy, refers to group therapy intervention; time, refers to pretest and posttest assessment; F, F-test value; p, significance; effect size, as measured by Cohen’s d; FAP therapy, fluent aphasic people attending therapy; FAP controls, fluent aphasic people not attending therapy; NFAP therapy, non-fluent aphasic people attending therapy; NFAP controls, non-fluent aphasic people not attending therapy; 1, first assessment (pretest); 2, second assessment (posttest). We bolded statistical significance (significant p level).*

Finally, there was a significant difference between the groups for the affectional expression in marriage in assessment of spouses of people with aphasia (Table 11). There was only one two effects showing interaction between time, aphasia’s type and therapy attendance [*F*(1,76) = 5.68, *p* = 0.020, $\eta_{\text{p}}^{2}$ = 0.02]. *Post hoc* analysis revealed significant (*p* < 0.001, *d*_*Cohen*_ = 0.42) decrease in emotional expression evaluations in FAP therapy group in time (from pretest *M* = 10.05, *SD* = 0.83, to posttest *M* = 7.50, *SD* = 2.16). Similar differences were confirmed for NFAPS therapy subjects. Their initial assessment of emotional expression in their relationship dropped significantly (*p* = 0.001, *d*_*Cohen*_ = 1.09) after 6 months from *M* = 10.10, *SD* = 1.29 to *M* = 7.90, *SD* = 2.53. Furthermore, first assessment of affectional expression provided by NFAPS control subjects was significantly (*p* < 0.001, *d*_*Cohen*_ = 1.50) higher than their second evaluation (pretest *M* = 9.80, *SD* = 1.05 and posttest *M* = 6.65, *SD* = 2.77).

**TABLE 11:** Two-way ANOVA and Bonferroni corrected *post hoc t*-test results for Dyadic Adjustment Scale affectional expression scores of spouses of people with aphasia.

	ANOVA					*Post hoc* comparisons		
	df	Mean square	*F*	*p*	Eta-square	Between group comparisons	*p*	Effect size
Aphasia type	1	1.06	0.184	0.669	0.002	FAPS therapy 1 vs. FAPS controls 1	1.000	
Therapy	1	6.01	1.046	0.310	0.013	FAPS therapy 2 vs. FAPS controls 2	1.000	
Aphasia type × therapy	1	6.01	1.046	0.310	0.013	NFAPS therapy 1 vs. NFAPS controls 1	1.000	
						NFAPS therapy 2 vs. NFAPS controls 2	1.000	

						**Pretest and posttest comparisons**		

Time	1	204.76	84.287	**<0.001**	0.526			
Time × aphasia type	1	6.81	2.802	0.098	0.035	FAPS therapy 1 vs. FAPS therapy 2	**<0.001**	0.422
Time × therapy	1	0.51	0.208	0.649	0.003	FAPS controls 1 vs. FAPS controls 2	0.624	
Time × therapy × aphasia	1	13.81	5.683	**0.020**	0.019	NFAPS therapy 1 vs. NFAPS therapy 2	**0.001**	1.095
						NFAPS controls 1 vs. NFAPS controls 2	**<0.001**	1.503

*Aphasia’s type, refers to fluent vs. non-fluent aphasia; therapy, refers to group therapy intervention; time, refers to pretest and posttest assessment; F, F-test value; p, significance; effect size, as measured by Cohen’s d; FAPS therapy, spouses of fluent aphasic people attending therapy; FAPS controls, spouses of fluent aphasic people not attending therapy; NFAPS therapy, spouses of non-fluent aphasic people attending therapy; NFAPS controls, spouses of non-fluent aphasic people not attending therapy; 1, first assessment (pretest); 2, second assessment (posttest). We bolded statistical significance (significant p level).*

## Discussion

Until now, most studies investigating therapy benefits and family relationship changes associated with experiencing aphasia derive from data gathered from caregiving spouses or other family members or do not consider aphasia’s type as a factor (Pound et al., 1999; Frosberg-Warleby et al., 2001; Martinsen et al., 2012; Northcott et al., 2016). Therefore we aimed to collate data from couples experiencing different communication problems and included repeated quality of marriage assessment as retrospective evaluation can be error-burdened. As we were interested in long-term therapy outcome related with dyadic relationship perception we incorporated a follow-up after 6 months.

Previous studies found that psychosocial intervention improves functioning during therapy and shortly after (Brown et al., 2010; Anderson and Whitfield, 2013). Consistent with this argument, but also in long term, participants of our study in the intervention group reported higher quality of marriage scores on posttest measures than controls, which were accompanied with reporting positive changes in marital adjustment dynamics over time.

Attending the therapy was associated with lasting higher evaluation of dyadic consensus and emotional expression in FAP group, as well as higher affection’s expression in NFAP group during follow-up. For FAPS subjects extended therapy benefits included the higher dyadic consensus. Unfortunately, over time and in spite therapy, NFAPS subjects still report decrease general quality of marriage evaluations as well as in consensus, satisfaction and emotional expression estimations levels. Nevertheless, lack of therapy intervention can lead to far greater changes in relationship. Aphasic people form control groups seem to notice a relationship crisis involving general dyadic adjustment and consensus (FAP) and emotional expression (FAP and NFAP). However, spouses not attending therapy suffer a decrease in general quality of marriage, consensus, emotional expression (FAPS and NFAPS), and satisfaction (NFAPS) levels over time.

What is interesting there are no significant changes in cohesion level irrelevant of therapy attendance or aphasia’s type. It is similar with satisfaction, but only from an ahasic person point of view, as only those evaluations seem constant. Those aspects of a relationship seems to be insusceptible to consequences of experiencing aphasia amongst older couples. This correspond with the fact mentioned by Goldstein (Draper and Blockheurst, 2007) that cohesion of couples in their later years does not suffer as much and is more resilient to crisis. It is partly due to a fact of fewer responsibilities carried by older couples. It is in accordance with our previous studies showing a protective value of previous marriage interaction between spouses with relationship duration over 14 years. It is more evident in aphasic patient’s perspective than in a caregiver’s point of view (Łapkiewicz et al., 2008; Orłowska, 2012).

We would like to emphasize, that when considering the marital adjustment changes dynamics within investigated groups, we observed no significant changes in perception of marital relationship amongst patients involved in therapy, irrespective of aphasia’s type. Their initial estimates do not waver and maintain rather positive and stable level. However, their spouses report decline in emotional expression (FAPS) or even general decline in time in overall marital adjustment, consensus, satisfaction, and emotional expression evaluations (NFAPS). On the other hand, marital relationship decline seems to be worse amongst control subjects, who were not involved in any kind of psychological support. In spite of initial non-distressed relationship they report deterioration of their bond in half a year’s time. All control subjects report decline in overall quality of marriage and consensus. These within-group changes have bigger size effects than in therapy patients groups. Additionally almost all control subjects report more problems in expressing emotions in their relationship. Satisfaction seems to suffer both in therapy and control NFAPS group.

Results of our study indicate, that applied group therapy program had a positive effect of preventing decline of quality of marriage. Though not all aspects of marital relationship can be maintained on the same level couples not involved in intervention experience deeper, more negative changes and are generally more distressed. This effect is strong for caregiving spouses and not in aphasic patients group. This corresponds with studies evaluating family support programs, which showed its benefit for social activities and quality of life improvement for carriers and no significant effect on patients (Mant et al., 2000).

According to our findings caregiving spouses perceive their marriage relationship as more strained if not offered support. This effect does not seem to be specific with aphasia’s type but perhaps can be explained by psychosocial burden, changes in life roles and other factors widely described in other studies (Visser-Meily et al., 2006). Furthermore, the general distress of caregivers of aphasic patients is not related to the aphasia itself but to personal resources of caregivers (North, 2007). Similarly their quality of marriage can be influenced more by understanding aphasia and receiving support during therapy.

Previous studies found, that although providing and discussing information on the illness, its consequences, are greatly appreciated by group therapy members, group intervention does not necessarily result in measurable improvements of relatives’ perceptions of personal, social, and familial burdens. Nevertheless, group therapy can lead to more realistic attitudes toward burdensome and severely straining situations and may help caregiving spouses with coping (Johannsen-Horbach et al., 1999). Studies demonstrated that merely providing information and recommendations on cognitive impairment in reducing the stroke survivor’s family stress (McKinney et al., 2002; Torres-Prioris et al., 2019). Pooled analysis of two individual psychoeducation programs provided by Cheng et al. (2014) showed a small effect on improving family functioning. Our study adds to that, as it provides evidence on preventive role of group therapy, which in long-term perspective safeguards against severe relationship deterioration.

Our previous studies indicate that quality of marriage seems to suffer more in couples struggling with fluent aphasia. The reported impairment is deeper and observed in more aspects including emotional expression, marital consensus, coherence, and satisfaction (Orłowska, 2012). Therefore the fact that spouses of people with fluent aphasia benefit more from education oriented therapy can be explained by the fact, that they need to understand the illness more, as it is not in agreement with common understanding of speech impairment. This is in accordance with studies indicating, that problems during communication situations can be attributed an inappropriate response by family and friends due to a poor understanding of the problem and exhaust caregiving spouses (Kagan, 1995).

Perhaps spouses of people with non-fluent aphasia do not benefit as much, because in their case knowledge and anticipating aphasia’s consequences does not help in coping as strongly and their relationship suffers more (Ellis et al., 2017). Therefore preventive role of this intervention is weaker.

Lastly, time factor has to be mentioned, as literature suggests most changes in quality of life and marital relationship surface after some time from the illness onset. Generally life of after-stroke couples seems to be affected after 4–6 months (Forsberg-Warleby et al., 2004).

Overall, current study showed, that there is a significant mean difference in quality of marriage between therapy attendants and controls, however, in half a year’s time those changes are diverse dependent on aphasia’s type and being a patient or a caregiver in a relationship. We also showed changes in dynamics of quality of marriage during this time in all investigated groups. As for aspects of marriage adjustment that profit from therapy it seems, that all its aspects benefit from therapy and do not drop due to experienced illness with the exception of cohesion, that remains rather constant.

## Conclusion

Based on our study results counselors are urged to be mindful of including relationship issues in therapy and rehabilitation process. There is a need to reach out to both aphasic and caregiving spouses and adjusting therapy goals and procedures to answer specific needs associated with aphasia’s type. Education oriented approach seems to be beneficial for their marital relationship and prevent it from deterioration.

This study utilized self-report data which rely on respondents perception and honesty. Also respondents are older Polish couples with marital relationship lasting over 15 years limiting generalization of findings to those in different locality age and shorter history of marriage. Furthermore, participants involved in intervention program were recruited amongst volunteers, who may have higher motivation and are more positive toward therapy in general. While recognizing the limitations of this study the results are significant to warrant additional research.

## Data Availability Statement

The datasets generated for this study are available on request to the corresponding author.

## Ethics Statement

The studies involving human participants were reviewed and approved by Ethical Committee, CM UMK, ul. M. Curie Skłodowskiej 9 85-094 Bydgoszcz. The patients/participants provided their written informed consent to participate in this study.

## Author Contributions

AR and EO participated in designing the study, acquisition of data, and analysis as well as approval of the final version.

## Conflict of Interest

The authors declare that the research was conducted in the absence of any commercial or financial relationships that could be construed as a potential conflict of interest.
